# *Fatty acid synthase 2* contributes to diapause preparation in a beetle by regulating lipid accumulation and stress tolerance genes expression

**DOI:** 10.1038/srep40509

**Published:** 2017-01-10

**Authors:** Qian-Qian Tan, Wen Liu, Fen Zhu, Chao-Liang Lei, Xiao-Ping Wang

**Affiliations:** 1 Hubei Insect Resources Utilization and Sustainable Pest Management Key Laboratory, College of Plant Science and Technology, Huazhong Agricultural University, Wuhan, 430070, Hubei, China

## Abstract

Diapause, also known as dormancy, is a state of arrested development that allows insects to survive unfavorable environmental conditions. Diapause-destined insects store large amounts of fat when preparing for diapause. However, the extent to which these accumulated fat reserves influence diapause remains unclear. To address this question, we investigated the function of *fatty acid synthase (FAS*), which plays a central role in lipid synthesis, in stress tolerance, the duration of diapause preparation, and whether insects enter diapause or not. In diapause-destined adult female cabbage beetles, *Colaphellus bowringi, FAS2* was more highly expressed than *FAS1* at the peak stage of diapause preparation. *FAS2* knockdown suppressed lipid accumulation and subsequently affected stress tolerance genes expression and water content. However, silencing *FAS2* had no significant effects on the duration of diapause preparation or the incidence of diapause. *FAS2* transcription was suppressed by juvenile hormone (JH) and the JH receptor *methoprene-tolerant (Met*). These results suggest that the absence of JH-Met induces *FAS2* expression, thereby promoting lipid storage in diapause-destined female beetles. These results demonstrate that fat reserves regulate stress tolerance genes expression and water content, but have no significant effect on the duration of diapause preparation or the incidence of diapause.

Diapause is a physiological process of programmed developmental and metabolic arrests that is often linked to enhanced tolerance of environmental stress in nematodes, crustaceans, insects, and fish^1^,^2^,^3^,^4^. In insects, diapause is a complex, dynamic, physiological process, with several distinct stages, including the diapause induction, preparation, initiation, maintenance, termination, and post-diapause quiescence^5^. Diapause in insects generally can last months to years^6^,^7^. Although a few insects continue to consume small quantities of food during diapause, most cease feeding^6^,^7^. Energy reserves accumulated during the diapause preparation stage are therefore critical to surviving diapause, and consequently can influence the decision to enter or terminate diapause, as well as post-diapause survival and fitness^7^. Lipids accumulated as fat reserves in the fat body provide a significant energy reserve for insects undergoing diapause^6^,^7^,^8^. Therefore, understanding the molecular mechanisms involved in lipid accumulation during diapause preparation is essential to understanding the metabolic processes that regulate diapause in insects^7^.

It has been demonstrated that fatty acid synthase (FAS) plays a central role in lipid accumulation in both vertebrates and invertebrates^9^,^10^,^11^. FAS regulates *de novo* lipogenesis by converting acetyl-CoA into palmitate, leading to the production and storage of triacylglyceride (TG)^12^. Upregulation of *FAS* was observed during early diapause in the mosquito *Culex pipiens*^13^,^14^ and knockdown of *FAS1* reduced lipid accumulation and affected the overwintering survival of females of this species^15^. *FAS* has also been found to promote lipid accumulation in another mosquito *Aedes aegypti*^9^. It is now generally accepted that FAS regulates lipid accumulation in insect diapause. However, exactly how this affects diapause remains unclear. For example, whether other important aspects of diapause, such as stress tolerance, the duration of diapause preparation, and the incidence of diapause itself, are regulated by FAS remains unknown.

It is widely recognized that energy balance plays an important role in stress adaptation and tolerance in invertebrates, including insects^16^,^17^,^18^, and that energy cost could constrain stress tolerance during diapause^17^,^19^. In addition, given the vital role of fat in diapause maintenance and post-diapause development^6^,^7^, we think it likely that lipid reserves could affect both the duration of diapause preparation and the decision to enter diapause. We suspect that FAS influences these aspects of diapause by regulating the process of lipid accumulation. Because absence of juvenile hormone (JH) is considered the primary reason for induction of reproductive diapause^20^,^21^, we think the high expression of fat synthesis-related gene, such as *FAS* in diapause-destined individuals is regulated by JH signaling.

The cabbage beetle, *Colaphellus bowringi* Baly, is an important pest of vegetable crops in Asia. The Xiushui population (29°1′N, 114°4′E) of this pest has two obvious occurrence peaks, one generation occurring in spring and one to three generations in autumn^22^. The larval stage is sensitive to temperature and photoperiod and the beetles enter diapause as adults in soil^22^. Newly emerged female adults begin to enter summer diapause after diapause preparation stage, namely, feeding for 4 days under 25 °C and Light:Dark (L:D) 16:8 h photoperiod. On the contrary is that females enter reproductive cycle after pre-oviposition stage, namely, feeding for the same number of days under 25 °C and L:D 12:12 h photoperiod^23^. The distribution of nutrients in diapause-destined and non-diapause-destined (reproductive) females differs. Non-diapause-destined females store yolk proteins in their ovaries for reproductive development, whereas diapause-destined females accumulate greater amounts of lipids (mainly TG) in the fat body^24^. These differences are apparent after newly emerged females had fed for just 2 days (under 25 °C), the peak stage of diapause preparation. This distinguishing characteristic makes *C. bowringi* an excellent model for studying the effects of lipid reserves on diapause. A search of our *C. bowringi* transcriptome database^25^ found two key lipid synthesis *FAS* genes, *FAS1* and *FAS2*. Our previous study suggested that the expression of *FAS1* were inhibited by juvenile JH signaling and the decreased expression of *FAS1* might block diapause in *C. bowringi*^26^. We suspect that by mediating lipid reserves *FAS* regulates stress tolerance, the duration of the diapause preparation stage, and the incidence of diapause in diapause-destined females.

To test this hypothesis, we quantified the transcription levels of *FAS1* and *FAS2* and used RNA interference (RNAi) to investigate the function of *FAS2* in diapause-destined females during the diapause preparation period. After silencing *FAS2*, the transcription of *heat shock protein (Hsp*) and oxidative stress-related genes, and the variation of water content were detected. In addition, we also determined the effect of silencing *FAS2* on the duration of diapause preparation and the incidence of diapause in diapause-destined females. Finally, we measured the effects of a JH analog and the JH receptor *methoprene-tolerant (Met*) on *FAS2* to investigate the regulation mechanism of *FAS* in *C. bowringi*.

## Results

### Sequence analysis of *FAS1* and *FAS2*

**Figure 1:**
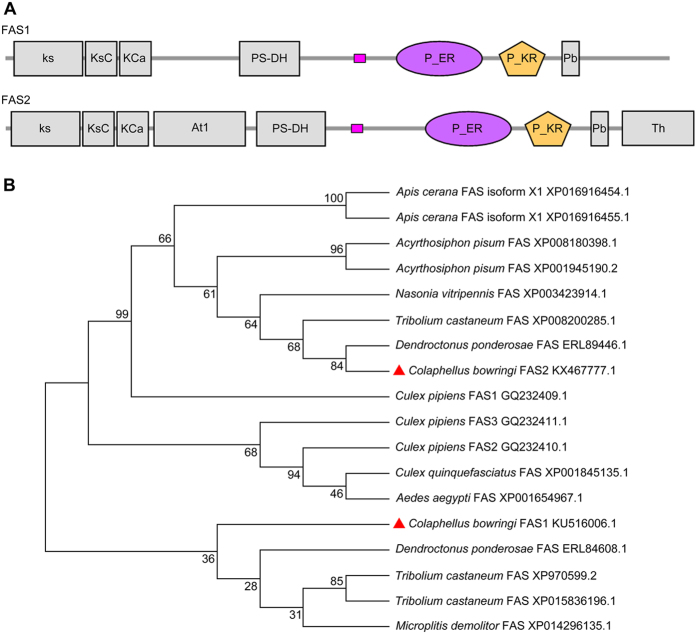
Gene identification of *C. bowringi FASs*. (**A**) Schematic diagram of deduced domains of FAS1 and FAS2. *FAS1* and *FAS2* protein sequences were deduced using the ExPASy Translate tool and protein domains predicted with the SMART tool. ks, ketoacyl-synt (pfam00109); KsC, Ketoacyl-synt_C (pfam02801); KCa, KAsynt_C_assoc (pfam16197); At1, Acyl_transf_1 (pfam00698); P_ER, PKS_ER (smart00829); P_KR, PKS_KR (smart00822); Pb, PP-binding (pfam00550); Th, Thioesterase (pfam00975). (**B**) Phylogenetic tree of evolutionary relationships between *FAS* proteins and their homologs in various insects. Red triangles denote *C. bowringi FAS1* and *FAS2* proteins.

Full-length *FAS1* and *FAS2* cDNA gene sequences were obtained from our *C. bowringi* transcriptome database^25^. These *FAS1* (KU516006.1) and *FAS2* (KX467777.1) sequences contained complete open reading frames of 6828 bp and 7134 bp, respectively, which encoded 2276 and 2378 amino acid proteins. Multiple alignment analysis of the FAS1 and FAS2 amino acid sequences indicated that they shared 39% amino acid identity, whereas FAS2 shared 78% amino acid identity with the FAS of red flour beetle *Tribolium castaneum* (XP_008200285.1) (Supplementary Fig. S1). On the Simple Modular Architecture Research Tool (SMART) website, the protein sequences of FAS1 deduced by the ExPASy Translate tool had seven functional domains, including ketoacyl-synt (ks, pfam00109), Ketoacyl-synt_C (KsC, pfam02801), KAsynt_C_assoc (KCa, pfam16197), PS-DH (pfam14765), PKS_ER (P_ER, smart00829), PKS_KR (P_KR, smart00822), and PP-binding (Pb, pfam00550). In contrast, the protein sequences of FAS2 contained nine functional domains, the above seven, plus Acyl_transf_1 (At1, pfam00698) and Thioesterase (Th, pfam00975) (Fig. 1A). A rooted phylogenic tree based on the protein sequences of FAS1, FAS2 and another 16 insect FASs was constructed in MEGA 4.1 using the neighbor-joining method. This placed FAS1 and FAS2 on two distinct, major branches (Fig. 1B). Although both FAS1 and FAS2 are most similar to other Coleopteran FAS, the *C. bowringi* FAS2 is actually more similar to the *C. pipiens* FAS1 than to the *C. bowringi* FAS1 (Fig. 1B). These suggest that FAS1 and FAS2 are likely to have different functions. Because Acyl_transf_1 domains in eukaryotic fatty acid synthase are involved in fatty acid biosynthesis^27^,^28^, we suspect that *FAS2* may be involved in fatty acid biosynthesis in *C. bowringi*.

### *FAS* expression patterns

**Figure 2:**
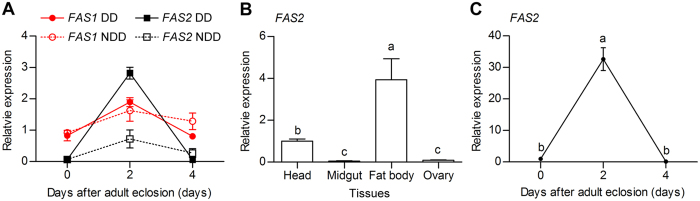
Expression of *FAS* genes in adult female C. bowringi. (**A**) Transcriptional expression of *FAS1* and *FAS2* in the entire body of adult female *C. bowringi* 0, 2, and 4 days after eclosion. DD, diapause-destined; NDD, non-diapause-destined (reproductive). Red branches present relative expression of *FAS1* and black branches present relative expression of *FAS2*. Values are expressed as means ± standard deviation (SD). (**B**) Expression of *FAS2* in different tissues of diapause-destined adult female *C. bowringi* 2 days after eclosion and (**C**) expression of *FAS2* in the fat body of diapause-destined adult female *C. bowringi* 0, 2, and 4 days after eclosion. The statistical significance of between-group differences was calculated using ANOVA, followed by the Tukey HSD test. Different letters indicate significant differences between groups (*P* < 0.05). All experiments were replicated three times.

To investigate the potential function of *FAS1* and *FAS2* in the diapause preparation period, we examined their transcript abundance at 0, 2, and 4, days after adult eclosion. Expression of both *FAS1* and *FAS2* peaked after female *C. bowringi* had fed for 2 days, the peak stage of diapause preparation. In detail, under diapause-inducing conditions, expression of *FAS2* was significantly higher than that of *FAS1* after female adults had fed for 2 days (*t* = −8.121, d = 4, *p* = 0.001) (Fig. 2A). What’s more, expression of *FAS2* in diapause-destined female adults was significantly higher than that of *FAS2* in non-diapause-destined female adults at the same timepoint (*t* = 11.395, d = 4, *p* < 0.001). These suggest that *FAS2* may be more important than *FAS1* in the diapause preparation stage. We then determined the relative expression of *FAS2* in the head, midgut, fat body, and ovary, of diapause-destined female adults that had fed for 2 days. *FAS2* mRNA was highly expressed in the fat body, but expressed at very low levels in the midgut and ovary (Fig. 2B). The temporal pattern of *FAS2* mRNA expression in the fat body of diapause-destined females was entirely consistent with that in the whole body. The mRNA level of *FAS2* increased significantly, peaking after females had fed for 2 days (Fig. 2C). Expression declined with continued feeding, decreasing to a very low level at the end of the diapause preparation stage. These results suggest that *FAS2* is mainly involved in the early period of diapause preparation.

### Silencing *FAS2* inhibits lipid accumulation in diapause-destined adult females

**Figure 3:**
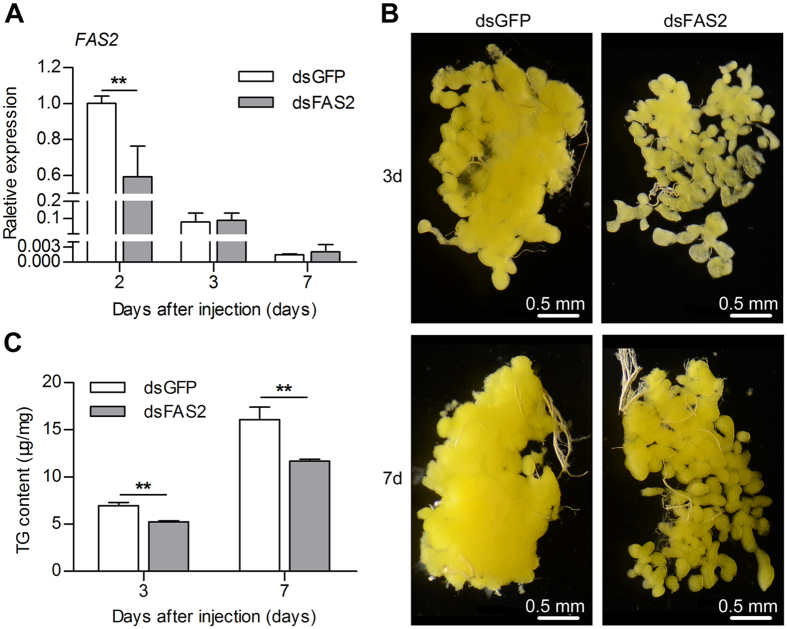
Effects of RNAi suppression of *FAS2* expression on *FAS2* expression (**A**), fat body morphology (**B**), and TG accumulation (**C**) in adult female *C. bowringi. FAS2* expression in the fat body was measured at 2, 3 and 7 days after dsRNA injection. The experiment was replicated three times and error bars indicate SD. The statistical significance of between-group differences was determined with an Independent-Samples *t* Test. **P < 0.01. Fat body morphology was observed and photographed at 3 and 7 days after dsRNA injection. TG content of entire body was measured at 3 and 7 days after dsRNA injection in three independent biological replicates, each comprised of five individuals. Error bars indicate SD. **P < 0.01 (*t* test).

In order to investigate its function, we silenced *FAS2* with RNAi in diapause-destined adult females during the diapause preparation stage. To verify the effectiveness of RNAi, we determined the transcript abundance of *FAS2* in the fat body at 2, 3 and 7 days after dsRNA injection. Expression levels of *FAS2* were reduced by approximately 40% compared to the dsGFP control after both groups had fed for 2 days (Fig. 3A). In addition, survival rates of both dsFAS2 and dsGFP groups were >96% after dsRNA injection. This indicates that knockdown of *FAS2* did not affect the survival of diapause-destined adult female *C. bowringi*.

We also observed the fat body morphology and TG accumulation after dsFAS2 injection. Photomicrographs show hypertrophy and clumping of the fat body in the dsGFP control group at 3 and 7 days after dsRNA injection. Hypertrophy of the fat body was, however, inhibited in the dsFAS2 treatment group in which the fat body became detached (Fig. 3B). These results suggest that suppression of *FAS2* inhibits the accumulation of lipids in the fat body. We used liquid triglycerides (GPO-PAP) method to further investigate the effect of suppression *FAS2* on TG accumulation. TG content in the dsFAS2 group was significantly lower than in the dsGFP control group (Fig. 3C), indicating that TG accumulation was reduced by the suppression of *FAS2*. These results indicate that *FAS2* promoted lipid accumulation during the diapause preparation stage.

### Silencing *FAS2* regulated stress tolerance genes expression and water content in diapause-destined adult females

**Figure 4:**
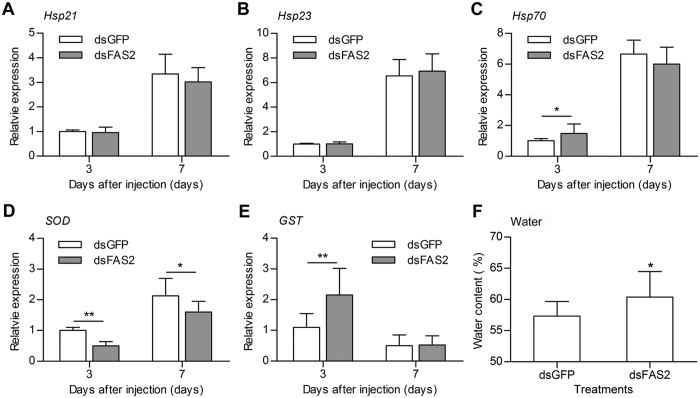
Effects of *FAS2* RNAi on the expressions of stress tolerance genes *Hsp21* (**A**), *Hsp23* (**B**), *Hsp70* (**C**), *SOD* (**D**) and *GST* (**E**), and water content (**F**) in adult female *C. bowringi*. The differential expression of stress tolerance genes was determined in the fat body at 3 and 7 days after dsRNA injection. Values are based on three independent biological replicates. Water content of diapausing adult female *C. bowringi* was measured at 7 days after dsRNA injection (dsGFP (control) n = 35, dsFAS2 n = 40). Error bars indicate SD. *P < 0.05, **P < 0.01 (*t* test).

To investigate the relationship between lipid accumulation and stress tolerance in diapause-destined female adults, we measured the expression of genes associated with stress tolerance and variation in water content after *FAS2* knockdown. We first measured the expression of three *Hsps, Hsp21, Hsp23*, and *Hsp70*, which are highly expressed in diapausing individuals and considered critical to stress tolerance during diapause^18^,^26^. There was no significant difference in the mRNA expression of *Hsp21* and *Hsp23* in the fat body between the dsFAS2 and dsGFP (control) groups (Fig. 4A,B). However, mRNA expression of *Hsp70* was upregulated in the dsFAS2 group after females had fed for 3 days (Fig. 4C). Because higher expression of *Hsps* occurred 7 days after RNAi, we deduce that the expressions of *Hsps* related to stress tolerance were not regulated by *FAS2*. We also examined the expression of another two diapause-related genes involved in oxidative stress, *superoxide dismutase (SOD*) and *glutathione S transferase (GST*)^29^,^30^. Interestingly, the mRNA expression of *SOD* in the fat body decreased, whereas that of *GST* increased after dsFAS2 injection (Fig. 4D,E). Water content is critical to the cold, or heat, hardiness of diapausing insects^31^,^32^. We found that water content significantly increased at 7 days after dsFAS2 injection (Fig. 4F). These results indicate that suppressing *FAS2* disrupted the stress tolerance mechanisms of diapause-destined adult females. We think that *FAS2* regulates stress tolerance genes expression and water content by regulating lipid accumulation in diapause-destined female adults.

### Silencing *FAS2* does not significantly affect the duration of diapause preparation, or the incidence of diapause

**Figure 5:**
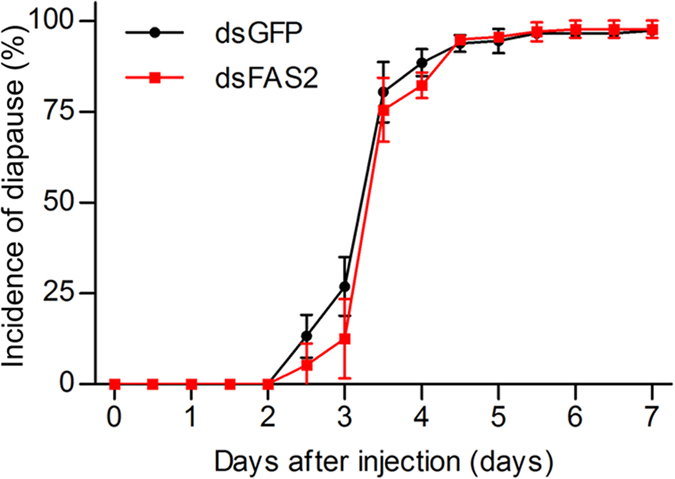
Effects of *FAS2* RNAi on the duration of diapause preparation and incidence of diapause in adult female *C. bowringi*. The time elapsed before adult female *C. bowringi* dug into soil to commence diapause was observed at 12 h intervals during the 7 days after dsRNA injection. Values are means ± SD based on three independent biological replicates. dsGFP n = 46, 49, 51; dsFAS2 n = 43, 46, 46. Log-rank (Mantel-Cox) test in Kaplan-Meier survival analysis was used to show the variation in the duration of diapause preparation between dsGFP (total n = 146) and dsFAS2 (total n = 135) groups.

*C. bowringi* digs into soil when they are about to enter diapause^22^. We recorded the number of individuals that dug into soil over a 12 h interval during each of the 7 days after dsRNA injection. There was no significant difference in the duration of the diapause preparation period between the dsFAS2 and dsGFP groups (Log-rank (Mantel-Cox) test, χ^2^ = 3.619, d = 1, *p* = 0.057) (Fig. 5). Meanwhile, *FAS2* suppression did not cause a significant difference in the incidence of diapause between groups (*t* = −0.297, d = 4, *p* = 0.782). These results suggest that suppressing *FAS2* can not delay diapause preparation, or affect the incidence of diapause in *C. bowringi* by decreasing lipid accumulation.

### *FAS2* transcript expression is regulated by JH-Met signaling

**Figure 6:**
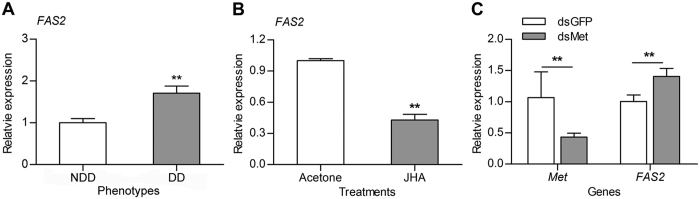
Transcriptional regulation of *FAS2* by JH-Met signaling. (**A**) Expression of *FAS2* in the fat body of non-diapause-destined (NDD) and diapause-destined (DD) *C. bowringi* female adults that had fed for 2 days. (**B**) The expression of *FAS2* in the fat body of diapause-destined females 24 hours after the injection of acetone (control) or a JH analog (JHA). Each diapause-destined female was treated with 15 μg of JHA on the day of eclosion. (**C**) Relative expression of *FAS2* and *Met* in the fat body of non-diapause-destined female *C. bowringi* 4 days after injection with either dsGFP (control) or dsMet on the day of eclosion. Values are the means ± SD of three independent biological replicates, error bars indicate SD. *P < 0.05; **P < 0.01 (*t* test).

qRT-PCR revealed that *FAS2* was highly expressed in the fat body of diapause-destined females compared to non-diapause-destined females after both groups had fed for 2 days (Fig. 6A). Because previous research demonstrated that JH signaling was significantly higher in non-diapause-destined females, and that JH-Met signaling inhibited the expression of *FAS1* to block diapause in *C. bowringi*^26^, we investigated the effects of JH on the expression of *FAS2*. The expression of *FAS*2 clearly decreased in the fat body of the diapause-destined females after these received a JH analog injection (Fig. 6B). Furthermore, *FAS2* significantly increased in the fat body of non-diapause-destined females after *Met* depletion, indicating that JH-Met signaling inhibited *FAS2* transcription (Fig. 6C). Overall, these results show that JH-Met signaling regulates the expression of *FASs* in *C. bowringi*.

## Discussion

During diapause preparation, insects need to store large amounts of lipids to provide energy during diapause and post-diapause development^6^,^7^. These accumulated lipids may also be important to the stress tolerance of diapausing insects^16^,^17^,^19^, but the effects of fat reserves on diapause remains unclear. For example, it is not clear if a regulatory network links accumulated lipids to other diapause-related features, such as stress tolerance, the duration of diapause preparation and the incidence of diapause. The results of this study show that the absence of JH-Met signaling induces *FAS2* expression, thereby promoting lipid accumulation in diapause-destined females. The lipid accumulation due to upregulation of *FAS2* regulates stress tolerance genes expression and water content, but has no significant effect on the duration of diapause preparation or the incidence of diapause.

Sequence analysis suggests that FAS1 and FAS2 share just 39% amino acid identity, and that FAS2 is more similar to *C. pipiens* FAS1 than *C. bowringi* FAS1. FAS2 has nine functional domains, seven of which are shared by FAS1, plus two others, namely Acyl_transf_1 (pfam00698) and Thioesterase (pfam00975). In addition, the results of our sequence analysis showed that three FASs of *T. castaneum* (XP015836196.1, XP970599.2, XP008200285.1) also had different domain architectures, and one of them had the same nine functional domains compared with *C. bowringi* FAS2 (Supplementary Fig. S3). However, two different FASs of *Bactrocera dorsalis* (JAC37527.1, JAC43906.1) had the same nine functional domains. These suggest that the different domain architectures are related with specific difference. These nine functional domains are common in the FAS protein family^28^. Acyl_transf_1 (pfam00698) domains occur in eukaryotic fatty acid synthase and are involved in fatty acid biosynthesis, whereas Thioesterase (pfam00975) domains often occur integrated in, or associated with, peptide synthetases involved in the non-ribosomal synthesis of peptide antibiotics^27^,^28^. We suspect, therefore, that FAS2 is involved in lipid biosynthesis in *C. bowringi*. In addition, our phylogenetic tree placed *C. bowringi* FAS1 and FAS2 on two deeply diverging branches, which suggests that they have different functions. As previously mentioned, *C. bowringi* FAS2 is very similar to *C. pipiens* FAS1, which has been reported to play an important role in lipid biosynthesis^15^. FASs regulate lipid biosynthesis mainly through being upregulated at the transcriptional level^33^,^34^. In female *C. pipiens, FAS* is highly up-regulated after the ingestion of a blood meal and is expressed sporadically as females enter diapause^13^. Our qRT-PCR results showed that *FAS2* transcript abundance was higher than that of *FAS1* in diapause-destined females at the peak stage of diapause preparation, suggesting that FAS2 plays more important role than *FAS1* during the diapause preparation stage. Consequently, we focused on investigating the function of *FAS2* in diapause-destined adult female *C. bowringi* during the diapause preparation stage.

The primary site of fatty acid synthesis, TG production and TG storage, in insects is the fat body^19^,^35^. We found that *FAS2* transcript abundance was significantly higher in the fat body than in other tissues of diapause-destined female *C. bowringi*. The observation that maximum expression of *FAS2* mRNA in the fat body occurred after females had fed for 2 days suggests that expression of *FASs* depends on the ingestion of nutritive substance. Silencing *FAS2* with RNAi caused an inhibition of the hypertrophy and clumping of fat body and a decrease in the total TG content of diapause-destined female *C. bowringi*. These results are consistent with the finding that fat storage is inhibited by RNAi-mediated suppression of *FAS1* in the mosquitos *A. aegypti*^9^ and *C. pipiens*^15^. Collectively, these results indicate that FASs generally regulate lipid accumulation during insect diapause.

Diapause is a state of developmental arrest during which insects are subject to different kinds of stress, including lack of food, reduced energy production, extremes of temperature, dehydration, and increased production of reactive oxygen and nitrogen species^16^,^36^,^37^. Accumulated energy reserves are essential to allow insects to withstand these various kinds of stress^17^. Hsps can increase the ability of insects to withstand environmental stress, including extremes of temperature, crowding, starvation, and hypoxia/anoxia, and are generally differentially expressed during diapause^18^,^38^,^39^,^40^. We speculated, therefore, that energy reserves, especially stored lipids, may promote *Hsps* expression during diapause. In a previous study, we found that three *Hsps* genes, *Hsp21, Hsp23* and *Hsp70*, were highly expressed in diapause-destined females compared to the non-diapause-destined females, and that the absence of JH-Met signalling in non-diapause-destined females can induce the upregulation of *Hsps*^26^. These findings suggest that *Hsps* play a role in stress tolerance and that JH signalling regulates their expression in *C. bowringi*^26^. However, that study did not determine how *Hsps* were upregulated in diapause-destined individuals. In this study, we investigated the possibility of link between lipid storage and *Hsps* expression. Our results show that *FAS2* knockdown had no effect on the expression of *Hsp21, Hsp23*, or *Hsp70*, in adult females after these had fed for 7 days after RNAi. This suggests that FAS2-regulated lipid storage may be not necessary for upregulation of *Hsps* expression during diapause. Although *Hsp70* was upregulated in adult females after these had fed for 3 days after FAS2 knockdown, we suspect that this was unrelated to stress tolerance because higher expression of *Hsp70* occurred 7 days after RNAi, when beetles had entered diapause. Since there is evidence that the absence of JH is essential for reproductive diapause to occur^3^,^26^,^41^, we suspect that JH-Met signalling may be the primary regulatory pathway for *Hsps* upregulation during diapause.

Antioxidant enzymes are critical for stress resistance during diapause^29^. Silencing *FAS2* in diapause-destined female beetles reduced expression of the *SOD* oxidative stress-related gene but increased that of *GST. SOD* expression was dramatically higher in diapausing *C. pipiens* females than in their non-diapausing counterparts^29^. However, *GST* was clearly downregulated in diapausing European corn borers *Ostrinia nubilalis* compared to non-diapausing controls^30^. This suggests that reduced lipid accumulation due to *FAS2* knockdown negatively affects the expression of oxidative stress-related genes in diapause-destined female *C. bowringi*. Because oxidative stress depends on the balance between pro- and anti-oxidants^30^,^42^, we couldn’t make inferences on oxidative stress from transcript levels of antioxidant levels alone. Thus, more experiment is needed to clarify whether oxidative stress was increased, decreased, or unchanged regulated by decreasing lipid accumulation. In addition, body water content is critical to surviving cold or hot conditions during diapause^31^,^32^. Low body water content is usually associated with high amounts of stored lipids in *C. pipiens*^31^. Our results show that silencing *FAS2* increased the water content of diapause-destined female *C. bowringi*, supporting the hypothesis that lipid accumulation reduces water content in diapausing insects. We think it likely, therefore, that increased water content reduce the ability of diapausing female *C. bowringi* to survive both cold temperatures and desiccation. Taken together, our results indicate that lipid accumulation makes contribution to both stress tolerance genes expression and water content, but has no obvious effect on *Hsps* expression. There are probably other factors, in addition to lipid accumulation, that affect stress tolerance in insects that need further investigation.

Because the energy reserves accumulated before diapause are critical for survival both during and after diapause, individuals must consume additional food to prepare for diapause^6^,^7^. We found that *FAS2* knockdown had no significant effect on either the length of the diapause preparation period, or the incidence of diapause. However, fewer beetles in the dsFAS2 group entered diapause after that had fed for 2–4 days than in the dsGFP control group (2.5 days, *t* = 1.639, *p* = 0.177; 3 days, *t* = 1.829, *p* = 0.141; 3.5 days, *t* = 0.702, *p* = 0.521; 4 days, *t* = 2.121, *p* = 0.101). We do not, therefore, rule out the possibility that more continuously effective RNAi could reduce accumulated lipids to an extent sufficient to delay the onset of diapause. There is evidence to suggest that the amount of lipids accumulated before diapause affects the lifespan of diapausing insects. For example, the lifespan of diapausing *C. pipiens* was significantly shortened after RNAi suppression of the forkhead transcription factor, possibly due to reduction in stored lipids^43^. However, the high variation in the lifespan of diapausing *C. bowringi*, from 5 to 38 months^22^, makes this question difficult to answer in this species. Alternative methods of detecting the effects of reduced lipid accumulation on the diapause maintenance are therefore required to test this hypothesis in *C. bowringi*.

JH is a strong candidate pathway for integrating the regulation of metabolic processes during both diapause preparation and diapause maintenance^7^,^26^. Consistent with the results of previous work on *FAS1*^26^, we demonstrated that *FAS2* was also regulated by JH-Met signaling, which suggests that this is a conserved feature in *C. bowringi*. These results indicate that energy reserves, especially accumulated lipids, can be regulated by the upstream endocrine system in diapause-destined *C. bowringi*.

**Figure 7:**
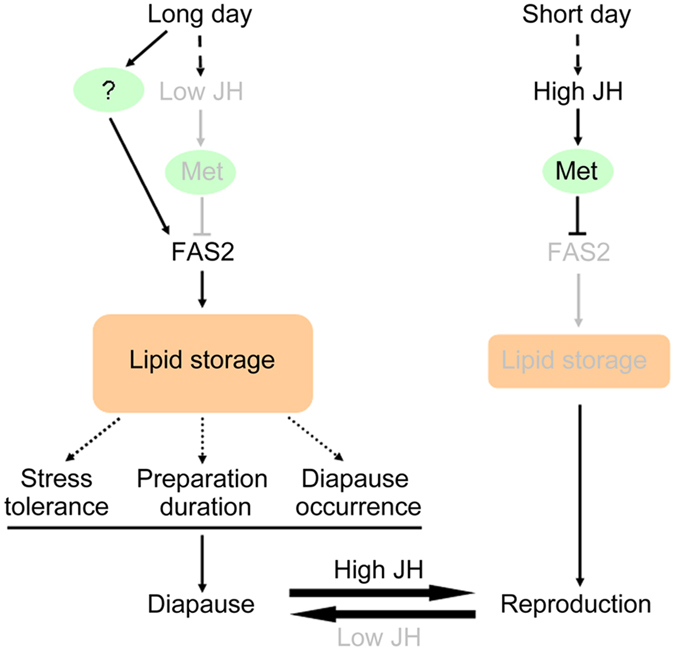
Model of how *FAS2* promotes diapause preparation in *C. bowringi*. Under short-day lengths (LD 12:12 h) at 25 °C, JH signaling is activated via Met and suppresses *FAS2* thereby inhibiting lipid storage and inducing females to become reproductive. Under long-day lengths (LD 16:8 h) at 25 °C, JH production is inhibited causing *FAS2* to be expressed. Large quantities of lipids are stored in the fat body which is essential for surviving diapause. Therefore, *FAS2* promotes diapause preparation by regulating lipid accumulation in adult female *C. bowringi*. The dashed lines suggest that the relationships between lipid storage and stress tolerance, preparation duration, diapause occurrence, are not well-founded, which need more experiment evidence. Components of the system that are less active or suppressed are shown in grey.

In conclusion, the results of this study show that *FAS2* promotes diapause preparation by regulating lipid accumulation and stress tolerance genes expression and water content in adult female *C. bowringi*. Based on these results, we constructed a model to describe the mechanism through which *FAS2* promotes diapause preparation in *C. bowringi* (Fig. 7). In this model, JH production inhibits the expression of *FAS2* via Met in non-diapause-destined female beetles. Diapause-inducing, long-day length conditions inactivate the JH-Met signaling pathway, thereby enabling *FAS2* expression and allowing diapause-destined female beetles to accumulate lipids. After silencing *FAS2*, downregulation of *SOD* and upregulation of *GST* as well as significant increase in water content, phenomena that have been linked to increased stress tolerance were observed. In future research, additional physiological experiments are required to conclusively show that accumulated lipids due to upregulation of *FAS2* can regulate stress tolerance. It has been reported that *FAS* gene transcription may be regulated by insulin through two transcription factors, upstream stimulatory factor (USF) and sterol regulatory element binding protein-1c (SREBP-1c)^11^. This leads us to suspect that increased *FAS2* transcription may be regulated by unknown upstream endocrine signaling factors in diapause-destined *C. bowringi*.

## Materials and Methods

### Insect rearing

More than 1000 cabbage beetles, *Colaphellus bowringi*, were collected as adults from a population in Xiushui County (29°1′N, 114°4′E), Jiangxi Province, China, and were transferred to the laboratory in late November 2008^44^. The offspring from post-diapause beetles were used in experiments and were reared as previously published work^44^. Diapause-destined female adults were obtained by rearing larvae at 25 °C under a light:dark (LD) 16:8 h photoperiod. Feeding for 4 days after adult eclosion is diapause preparation stage. Non-diapause-destined, namely reproductive, females were obtained by rearing larvae at 25 °C under a LD 12:12 h photoperiod^26^,^44^,^45^. The 0, 2, 4 days of newly emerged adults feeding are the early, peak, late, stages of diapause preparation or pre-oviposition stages.

### cDNA cloning and sequence analysis

*FAS1, FAS2, SOD* and *GST* sequences were retrieved from the *C. bowringi* transcriptome database^25^. The cDNAs of these genes were isolated using the corresponding primers (Supplementary Table S1), inserted into a T vector using the pMD – 18 T Vector Cloning Kit (Takara, Japan), and directly sequenced. The amino acid sequences of these genes were deduced using the ExPASy Translate tool (http://web.expasy.org/translate/). Gene identifies were confirmed using MEGA 4.1 software and amino acid sequences were used to construct rooted, neighbor-joining phylogenic trees (Fig. 1, Supplementary Figs S1 and S2). The protein functional domains of FAS1 and FAS2 were predicted using SMART (http://smart.embl.de/) and NCBI Conserved Domain Search website (https://www.ncbi.nlm.nih.gov/Structure/cdd/wrpsb.cgi). *FAS1, Hsp21, Hsp23* and *Hsp70* sequences were the same as those previously used by Liu *et al*.^26^. All *C. bowringi* cDNA sequences obtained are available from GenBank under the following accession numbers, KX467777.1 (*FAS2*), KX467778.1 (*SOD*), KX467779.1 (*GST*), KU516006.1 (*FAS1*), KU516010.1 (*Hsp21*), KU516011.1 (*Hsp23*) and KU516012.1 (*Hsp70*).

### Quantitative Real Time PCR (qRT-PCR) for mRNA quantification

cDNA was synthesized and mRNA levels of selected genes quantified as described previously^25^,^26^. Total RNA was extracted using RNAiso Plus (TaKaRa Bio., Dalian, China) following the manufacturer’s protocol. RNA concentration and purity were determined with NanoDrop 2000 (Thermo Scientific, Wilmington, DE, USA). RNA integrity was verified by electrophoresis in 1% native agarose gel. One μg of total RNA was used to synthesize first-strand cDNA using a PrimeScriptRT reagent kit with gDNA Eraser (Perfect Real Time) (TaKaRa Bio, Dalian, China), according to the manufacturer’s protocol. The synthesized cDNA was stored at −20 °C until required.

Quantitative PCR reactions (qRT-PCR) were performed on a template of 20-fold dilutions of the cDNAs and SYBR Premix Ex Taq II (TaKaRa, Dalian, China) using a MyIQ2 Two-color Real-time PCR Detection System (Bio-Rad, USA). The qRT-PCR primers were designed using an online tool (http://www.ncbi.nlm.nih.gov/tools/primer-blast/) (Table 1)^26^. *Ribosomal protein L19 (RPL19*) was used as the reference gene for normalizing gene expression according to previously published work^25^. qRT-PCR data were collected from three independent biological replicates, each of which was analyzed with three technical replicates of the 2^−ΔΔCT^ method^46^.

### RNAi of FAS2 in C. bowringi

A 512-bp fragment of the *FAS2* gene was amplified by PCR with the corresponding primers (Table 1), verified by direct sequencing, and then purified with phenol-chloroform. Approximately 1 mg of the DNA template was used to produce dsRNA against *FAS2* (dsFAS2) with a T7 transcription kit (Fermentas, Lithuania), according to the manufacturer’s instructions. dsRNA against *green fluorescent protein* (dsGFP) was synthesized similarly to serve as a control. dsRNA integrity was verified with electrophoresis in 1% native agarose gel, and the concentration was measured with NanoDrop 2000 (Thermo Scientific, Wilmington, DE, USA). One μg of dsRNA in 200 nL was microinjected into adult female beetles on the day of eclosion (day 0) before they had commenced feeding^26^. Total RNA was then extracted from the fat body after 2, 3, and 7 days to determine the effectiveness of RNAi. Survival rates were determined 7 days post-injection.

### Phenotype analysis in the fat body after RNAi

Fat bodies were removed 3 and 7 days after dsRNA injection^47^ and photographed with a digital camera (Nikon D5100, Nikon Imaging (China) Sales, China) mounted on a stereo-microscope (SMZ-t4, Chong Qing Optec Instrument, China).

### TG content

Total TG content was determined using the liquid triglycerides (GPO-PAP) method^48^ performed using a Triglycerides Assay Kit (Nanjing Jiancheng Institute, China) 3 and 7 days after dsRNA injection, according to the manufacturer’s instructions. A pool of 5 beetles was homogenized in 1 mL of ethanol and centrifuged at 6000 rpm for 5 min. The supernatant was transferred to a new tube and the volume increased to 1 mL with ethanol. 2.5 μL of the supernatant was added to 250 μL of the reaction solution, incubated at 37 °C for 10 min, and the absorbance detected at 500 nm. There were three independent biological replicates for each treatment, each of which was analyzed with three technical replicates.

### Water content

The water content of adult females was measured 7 days after dsRNA injection. The weights of 35 diapausing adult females in the dsGFP control group, and 40 diapausing adult females in the dsFAS2 treatment group were recorded. Weighed individuals were then dried at 80 °C for 24 h and reweighed to calculate their water content by subtraction.

### Duration of diapause preparation and incidence of diapause

The number of females that entered diapause was recorded at 12 h intervals over a 7 day period following dsRNA injection. This process was replicated three times, each replicate was comprised of 43–51 individuals. The estimated incidence of diapause was the average of these three replicates.

### Transcriptional regulation of *FAS2* by JH-Met signaling

Before investigating the regulation of *FAS2* by JH-Met signaling, the expression of *FAS2* was determined in the fat body of both non-diapause-destined and diapause-destined female *C. bowringi* that both had fed for 2 days. Diapause-destined female adults were treated with either acetone (control) or 15 μg of JH analog methoprene solution (Sigma-Aldrich, St Louis, MO, USA) on the day of eclosion (day 0), before they had commenced feeding^26^. The total RNA of the fat body was extracted 24 h later and *FAS2* expression analyzed using cDNA synthesis and qRT-PCR. Non-diapause-destined females were treated with either dsGFP (control) or dsRNA against *Met* (dsMet) on day 0, and *Met* and *FAS2* expression in the fat body measured 4 days later^26^. These experiments had three independent replicates, each of which was comprised of the fat bodies of 15 individuals.

### Statistical analysis

Statistical analyses were conducted in SPSS 11.5 (SPSS Inc., Chicago, IL, USA). Results are presented as the means ± standard deviation (SD). One way ANOVA followed by Turkey’s HSD multiple comparison tests was used to compare the expression of *FAS2* in different tissues and on different days (*P* < 0.05). Log-rank (Mantel-Cox) test in Kaplan-Meier survival analysis was used to show the difference in the duration of diapause preparation between dsGFP and dsFAS2 groups^49^. The significance of differences between experimental groups in remaining experiments was assessed using Independent-Samples *t* Test (*P < 0.05, **P < 0.01).

## Additional Information

**How to cite this article:** Tan, Q.-Q. *et al. Fatty acid synthase 2* contributes to diapause preparation in a beetle by regulating lipid accumulation and stress tolerance genes expression. *Sci. Rep.* **7**, 40509; doi: 10.1038/srep40509 (2017).

**Publisher's note:** Springer Nature remains neutral with regard to jurisdictional claims in published maps and institutional affiliations.

## Supplementary Material

Supplementary Information

## Figures and Tables

**Table 1:** Primer sequences for qRT-PCR and dsRNA synthesis.

Genes	Forward primers (5′-3′)	Reverse primers (5′-3′)	E (%)	R^2^
qRT-PCR				
* RPL19*	gtaatgcgatgcggcaagaa	gagtgcaccgctacaggttt	102.2	0.998
* FAS1*	ggccaacgagttatgggtat	agggcataaacagtcgttcc	90.3	0.992
* FAS2*	cactgaactctgccacgtct	cgcgtttgccactagaatcg	92.5	0.991
* Hsp21*	acgtcaggtcctggagatca	tgaactgctgaacgtcgaga	98.0	0.990
* Hsp23*	tgtactgagaccgctgagga	gtagcgaggcctgtttggta	90.7	0.988
* Hsp70*	tgactttcgatctggacgcc	gtcgatatcctgctgcgaca	93.8	0.990
* SOD*	aagctgggacatatagcgcc	gcctaggtctccaacgtgtc	98.2	0.996
* GST*	tcgaaatacgccaaagggca	tctcagtggttccaacaggc	104.1	0.994
* Met*	caattgctcaacacccagcc	ccttcgttgagcgacagtct	98.2	0.998
RNAi				
* GFP*	gcgtaatacgactcactataggtggtcccaattctcgtggaac	gcgtaatacgactcactataggcttgaagttgaccttgatgcc	n.a.	n.a.
* FAS2*	gcgtaatacgactcactataggaagaacacaaccttccacgg	gcgtaatacgactcactataggccgcttctggaagtgagaac	n.a.	n.a.
* Met*	gcgtaatacgactcactataggatgattgaggaagtgtcggg	gcgtaatacgactcactatagggattctcgtggtggaccagt	n.a.	n.a.

E, PCR efficiency; R^2^, Standard curve R^2^; n.a., not applied.
