# Supplementary material for: Fatty acid synthase 2 contributes to diapause preparation in a beetle by regulating lipid accumulation and stress tolerance genes expression
Source: Sci Rep. 2017 Jan 10;7:40509. doi: 10.1038/srep40509 (PMC5223116; doi:10.1038/srep40509)
Supplement: Supplementary Information [file srep40509-s1.pdf]

Fatty acid synthase 2 contributes to diapause preparation in a beetle by regulating lipid accumulation and stress tolerance genes expression

Qian-Qian Tan, Wen Liu*, Fen Zhu, Chao-Liang Lei & Xiao-Ping Wang*

Hubei Insect Resources Utilization and Sustainable Pest Management Key Laboratory, College of Plant Science and Technology, Huazhong Agricultural University, Wuhan 430070, Hubei, China.

* Co-corresponding author

Correspondence: liuwen@mail.hzau.edu.cn or xpwang@mail.hzau.edu.cn

Additional information in this file.

Figure S1. Alignments of *C. bowringi* FAS protein sequences.

Figure S2. Gene identification of *C. bowringi SOD* and *GST*.

Figure S3. Schematic diagram of deduced domains of FASs of *Tribolium castaneum* (A) and *Bactrocera dorsalis* (B).

Table S1. Primers used in cloning *C. bowringi* cDNAs.

Figure S1

```
CbFAS1 KU516006        : MTRINLDRDRKNQYTEYG----RILAHPPPSEAVVISGISGVFPKCRNVEELRDSLMNKLDLTTAHYREG-FDHAELPKRSGTLNTGLQDFDAGFFGMHENQADPCSIETRILLEKSIEAIIDAGMNPK : 124
CbFAS2 KX467777        : MPAR-------APEPER-------HLGNSFSDDVVITGISGRLPESNTIEEFKQQLFDGVDLVTDDERRWPSGLYGLPTRTGKIKN-LQYFDATFFGVHAKQANVMDPQLRMLLELTHEAIVDAGMNPA : 114
TcFAS XP_015836196.1 : MSRS------SEPNHQFS----RWLSQPPEGDEVVVTGMSGRLPDSLHLHHFQDNLFNKVEMVSDDDRRWKLDHKEIPQRTAKILD-IDKFDTGFFGLHFRQANTMDPMIRMVLESAMEAIFDAGVNPR : 118
TcFAS XP_008200285.1 : MPAR-------VPDAQNG------HHVNAISDDIVITGISGRLPESSNIEEFKQQLFDGVDLVTDDARRWPSGLYTLPARTGKLKD-LSRFDATFFGVHHKQATVMDPQLRLLLELTYEAIVDAGVNPN : 115
AiFAS AGR49310.1      : MPSA-------VTNGAR-----------GSEDDIVLTGLSGRLPESDTIEEFAQQLFDGIDLVTADDRRWTPGLHGLPERNGKLKD-LAHFDATFFGVHAKQAHLMDPQLRLLLELTHETIIDAGINPS : 110
ApFAS XP_008187030.1 : MSRQQAIELKIKTDREIALRAGRRLVKCDPGDEIVISGFSGSFPNSDGLPDFADNLFNKVDLISDDNRRWTIEHPEIPQRTGKLNQ-VNKFDAAFFGVHYKQAHTMDPMCRIILEKSYEAIVDSGNSPK : 128

CbFAS1 KU516006        : DIESTKTGFYLGYCYSDTESTLGWKTHKEKRFPILTAARFSMVSRVAYFLKLKGPTYQCDSACSSSLHALDHAFKDIRSGECENAIVAGANLSMFPGTTLQFAKLGVLSPRGICQPFDKNGDGYVRSEV : 253
CbFAS2 KX467777        : ELRGSKTGVFIGVSDSESSEFWTQDPEKVNGYGLTGCCRAMFPNRISYTFDLTGPSYAIDTACSSSLFAFQQAITAIKTGQCDAAIVGGVNLLLKPTSSLQFHRLSMLSPQGMCKAFDASGNGYVRSEA : 243
TcFAS XP_015836196.1 : ELEGTRTGVFISASWSDMENRTLTSISEPERYGITGYLRSLLAQRISYYLKLKGPSFIVDTACSSSGNALEHAFKAIRSGQCDQALVGCSNLLLHPGNSLQFFRLGVLSSEGKCRVFDQDASGYVRGET : 247
TcFAS XP_008200285.1 : DIKGSKTGVFIGVSDSESSEYWTQDPDKINGYGLTGCCRAMFSNRISYTFDFSGPSYALDTACSSSLVALQQAITAIKTGQCDAAIVGGVNLLLKPTSSLQFHRLGMLSPQGMCKAFDASGNGYVRSEA : 244
AiFAS AGR49310.1      : ELRGSRTGVYVGVSNSETEEMWTVDPDKINGYALTGCCRAMFPNRISYTFDLKGPSFAVDTACSSSMFALAQAATAIRSGHCDAAIVAGCNLCLKPANSLNFHRLSMLSPEGRCAAFDASGRGYVRSEA : 239
ApFAS XP_008187030.1 : SLKGSKTGVFVGACFSESEKTWFYEKLQVNGFGLTGCARAMLANRISYWLGVNGPSYSVDSACSSSLYALEHAYKAIRDGHCDSALVGGCNLCLHPYVSLQFARLGVLCADGRCKSFDEAANGYVRSEA : 257

CbFAS1 KU516006        : ITVVLLQKARNSKRIYAQVINTKCNSDGFKDNGITFPSRAAQAELIAEVLEESNVQPDNVTYLEGHATGTSAGDPEECAAIDSVIGQ-NRKTPLRVGSVKSNMGHSEPSAGICSIVKCLISMESGFLFP : 381
CbFAS2 KX467777        : AVVVYLQKASAARRVYATVLGAKTNTDGNKEQGITFPSGQMQNKLIREVYEEIGVKPQEVSYVEAHGTGTKVGDPQEVNSIADFFCK-NRKDPLLIGSVKSNMGHSEPASGLCSLAKIVIAMEAGMIPQ : 371
TcFAS XP_015836196.1 : IACVFLQKAKNSRRIYAQIINAKINSDGFKEQGITFPSTQAQKILMTEIYDESNVHPNQLSYLEAHGTGTQVGDPQEVEAIDCALAK-PRNKTLLVGSVKSSIGHTEPASGICSLIKVLLAMESGLISP : 375
TcFAS XP_008200285.1 : AVVVFLQKASVAHRVYATVLGAKINTDGYKEQGITFPAGPMQNQLIRETYAEFGVDPNEVSYVEAHGTGTKVGDPQEVNSIADFFCTKNRKEPLMIGSVKSNMGHSEPASGLCSLAKIVIAMESGMIPG : 373
AiFAS AGR49310.1      : AVAVLLQRRGAARRVYATLRGLRVNTDGAKDQGITFPSGDMQRRLAEETFAEAKLRPADVAYVEAHGTGTKVGDPQEVNAIAELFCK-GRKGPLLLGSVKSNMGHSEPASGLCSVAKVVVAMERGIIPS : 367
ApFAS XP_008187030.1 : VCVLYLQKAKNAKRVYAKVVHAKTNCDGYKEQGITYPSGPLQQRLLEEFYEECEIKPSDLAWIEAHGTGTKVGDPEEVKALENVFCP-GRTTPLLIGSVKSNIGHSEPASGLCSVAKVIIAMESGFIPP : 385

CbFAS1 KU516006        : TINYSETRPGISSILEGRLEVVTEPIKITEEHPLMAVNSFGFGGSNSLSLIRSISKKKQNGGEPSDNLPRLVMVSGRNLEAVNSLLDSVPSNPLDAEYIRLLQNSFRIDIPNNSFKGYMIISKTTEISR : 510
CbFAS2 KX467777        : NLHFKSPNKDIPALNDGRLKVVAKNEPWNGG--IIAINSFGFGGANAHVVLRSNPKPKTLWPVG--PIPRVVGVSGRTQEAVTNFLAKVEQNKNDEEFLALLDEIHSKNINGHAYRGYVLLKDQ-AVKE : 495
TcFAS XP_015836196.1 : NINLKRIKEGMVGFEKQRMKAVTEITELEGNEAIVGINNFGFGGNNCHAIVKRFGKMKKNGGFPDDDLPRLVCVSGRSEEAVAQILNQVN-LKLDVEFNALLHEIYKKDIPNHMFRGFTLVGKSGPLVT : 503
TcFAS XP_008200285.1 : NLHFKSPNTDIPALLDGRLKVVAKNEPWKGG--LIGVNSFGFGGANAHIILKSNPKPKASWPTD--PLPRVVGVSGRTPEAVNTFLDHVEKNKNDEEFLALIDQIHKSNITGHAYRGFTVLSNP-ALRE : 497
AiFAS AGR49310.1      : NLHYKNANPDIPALSDGRIKVVDRNTPWDGG--LVAINSFGFGGANAHVIFESEAGGGAARTPARYAAPRLVLASGRTEEAVQELTQLAAQHREDAGLHALLDAVHRHNIPGHSYRGFAVLSDP-PVQE : 493
ApFAS XP_008187030.1 : NINFNTPREDITAFYNGRINVVADKTPWNGG--LVGINSFGFGGANCHVLLNWNNKSKINNGLPEDDLPRLVVASGRTEEAVTTILNDFLSRSLDKEYVRLLQDVQAEQIPGHIYRGYTIIKKDCKANQ : 512
```

To continue the last alignment

```
CbFAS1 KU516006         : SFDKI--SLERLPLYFVFGDLR-DWYALLQQFSAVPVFAGSLQNIQGYLKKKDVDVKTTILSADEEVR--KSYNTLGSLVIQICLADVLKKLNLRPVSAIGYSFGELVCAYYDDDLDLETVLGLCLILN :  634
CbFAS2 KX467777         : VTQLP---GENRPVAFVFSGMGSQWPGMAKDLMQLDCFKNSIIKCSDALKPHGINLEDILINGTEATFDNVLNSFISIAAMQVALTDVLKTLGIEPDVIVGHSVGEVGCAYADGTFTGEQAVLAAYSRG :  621
TcFAS XP_015836196.1 : SVKLR--RALDTPFYVYFGQFG-RNYKLGQFLMGFPVFCDTIKRIN------------ETLSGKTPPQ--AYQPILGSLIVQLGLVDLLKALNLSPV-IYGDNWGKLATAYYYKTLTLEECI------- :  607
TcFAS XP_008200285.1 : VTEVS---NEKRPVWFVFSGMGSQWPGMAKQLMEVEVFRNSIKRSAEILKPHGINLEDIIVNGTEATFENVLNSFVSIATMQVALTDVLKALEIEPDGIVGHSVGEVGCAYADGTFTAEQAVLAAYARG :  623
AiFAS AGR49310.1       : CIEIES--GDPRPVWFVFSGMGSQWPGMAKTLMQLPAFAASINRSAAALRPHKLDLINIITDAPAAAFDDVINSFVSIAAVQVALVDVLRALEIRPDGIVGHSVGEIGCAYADETLTAEQAVLAAYWRG :  620
ApFAS XP_008187030.1 : SMDLQYYSGESRSVCFVFSGMGSQWTGMGTSLMQLPIFNESISKSHSILKEFGIDLVKIITSTDANILNNTVNSFVGIAAMQIALFDVLVAIGITPDIIIGHSIGELVCAYADGCLTSEQTIKVAYYYG :  641

CbFAS1 KU516006         : GEAIFNGEKKNGLIFG-------------------SSSTNEKTLKIRKDIVTKFSEELKNKQT-----------------------------------VKRSEAFVDAILRLN---------------- :  693
CbFAS2 KX467777         : RAILESKLPLGAMAAVGLSWEEVKRRCPPEIYPACHNSEDSVTISGPPAAIEKFVAELQAQDIFAKAVKSSGTAFHSKYIADAGPKLRKALDEIIPNPKPRSPRWISSSIPESGWGTPMAQHSSAAYHV :  750
TcFAS XP_015836196.1 : -----------------------------------ISATNN------HGKTPDFAPELPNGQ-------------------------------------LRSDSLTHDTLLN----------------- :  641
TcFAS XP_008200285.1 : RAIIESKLIPGQMAAVGLTWEEAKRRCPPDVFPACHNSEDNVTISGPLDAVNKFVDQLTSENIFAKKVNSSNIAFHSKYIAEGGPKLRKALEAIIPNPKPRTSRWISSSIPESAWGTPLAQQSSAAYHV :  752
AiFAS AGR49310.1       : RSIVDAKLAPGAMAAVGLSWEQCEARCPPDVVPACHNANDSVTISGPVDSLEKFVAELSAEGTFARRVNSSGVAFHSKYIAAAAPLLRRSLEKVITAPKPRTSRWVSSSLPRDQWNSDLAKLSDANYHV :  749
ApFAS XP_008187030.1 : LATLNSKIPLGAMAFVGIGYNQIKDLLPTNVVVAWHNSPDSCAISGLKESVEQFVLKLKSKDISTQMINVLNTPYHSTSIKKAIPSLLEYLKNIITNPKLRSGKWLSTSVPEEKWGEDKAKYCSAEYCA :  770

CbFAS1 KU516006         : --------ITSTHDIIHSDSLIVEIGEN----ELMNYDS-EK--TPWELKSLSFHSFIEFLQ-ILGSLYILGYNASIENLYPTIEFPVSKSTPNISSKIKWNHERKWFIHSQMVLNAKKYAPRHFEIDL :  806
CbFAS2 KX467777         : NNLLSPVLFHEALKHIPENAVVIEIAPTGLLQAILKRAL-GPKVTNVSLVKRGHADNIEFLLSALGKIYNAGAQPKLSNLYHPVSFPVGRGTPMINSMIEWDHSIEWSVANFSGKGSRS-GELVVDIDL :  877
TcFAS XP_015836196.1 : ----------------LKNAFILNVSDS----NLNNRD-----------VMITDGIKINFLE-TIGRLYELGHTPQLNQLYPKVEFPVSRGTGMISPLVKWNHDRSWYTYKFREFTITDAEQRDYFISY :  738
TcFAS XP_008200285.1 : NNLLSPVLFHEALKHVPENAIAIEIAPAGLLQGILKRAI-GPKSTNISLVKRMHENNVEFLTSAIGRIYNAGAQPKVGNLYHPVSFPVGKGTPMIASMIQWDHSTEWAVANFCDKGSRS-GELVIEVDL :  879
AiFAS AGR49310.1       : NNLLSPVRFADALREVPARSIVVEVAPHALLQAVLKRALPAPAAAHVPLVRRDAACACAHLLAAAGRLYAAGAQPAVGRLYPAVVWPVPRGTPGLASRVRWDHRLEWQVAHFG-NASRS-GENVIEYDV :  876
ApFAS XP_008187030.1 : NSLLNSVLFDETFEHVPKGSVLIELAPHGVLQDVLNRSH-KTNITNVDLASRNYKDGIDYLLSAFGKIFEAGLNPKISNLYPDIEFPVSRGTPMIAPLVRWEHSEDWYVTMYRVQDKIKSGERNISISL :  898

CbFAS1 KU516006         : KNKQWSFVAGHLIDGRLLFPASGYLYLAWQTLLGKFPKLMLEYTVTFRNCRFIRATNVPLDGSVALDVTIQP-SGHFEVLEGESIVVAGEIT----IEQREKAIERLPV--IPSDSNMMNSKDIYKELR :  928
CbFAS2 KX467777         : SKESDQYLVGHTIDGRVLFPATGYLTLVWKTFAKLRNEDFEQLPVILEDVQFHRATIMPKEGSVKFLINIFEGTGEFELCEGGSVAVSGKISVPEDVS-KEALDLPKPHGKTEKDVLPLNSADVYKELR : 1005
TcFAS XP_015836196.1 : VYDEYKFMQGHVIDDRNLFPATKYLTLVWETFAQSRRLLPEDVSVVFENCRFVRAVTMPKTGFVKLLVTIGLGNGNFEVLEKDTLVVSGKIRTLEETNQKETDFGPIDL--GSDCTQILEHDEIYRELY :  865
TcFAS XP_008200285.1 : SKEDDQYLAGHAIEGRVLFPATGYLTLVWKTFAKLRNEDYEQLPVIIEDVQFHRATIMPKEGTVRFLINIFEGTGEFELCESGSVAVSGRISVPEDVS-KELLHLPKPTPEVTKDLLPLNTADIYKELR : 1007
AiFAS AGR49310.1       : SRNDDSFITGHNIDGRVLFPATGYLTLVWRTMAKLNNRKPEETPIVMENIQFRRATIVSRDTPVRFLINVLDGTGEFDVCEGGAVVVTGTVRLADDPAGERLKDLDCTPPRREDGLLPLVTDDIYKELR : 1005
ApFAS XP_008187030.1 : KDEEHEYLSGHVIDGRNLFPATGYLVMAWETLALMRGELYSEVPVVFENVRFQRATNIPKDGNVEFIVMVQKGSGTFEVVESGAPVVTGRLYIPTDANYEMIDMPPHPD--EPSDTDLN-IKDIYKELR : 1024
```

To continue the last alignment

```
CbFAS1 KU516006        : LRGYNYNGEFQAIREVNLDATLAHITWNDNWITFIDNMFQMKILKSDSRLLYVPTFIAKITIANKYHERFVAGLLGEKSGVLPVHYYPEVDTIRCGGIEVHDLKASSIPRK-KVTATPVLETYQFLPN- : 1055
CbFAS2 KX467777        : LRGYDYGGIFRGITESDNWGISGKLKWENNWISFIDTMLQFSILGQDTKELYLPTRLQRAFINPKEHLKAISELRESE--GVPVHMYRNIGVIKSAGIELRGMKASLAPRRQQSQAPPKLEKYQFVPYE : 1132
TcFAS XP_015836196.1 : LRGYNYSGLFKGIAKCNVEGSSGLVKWEENWCTFTDKMLQLKILQNDTRLLYVPVGIQKMVINPVKHKKFAEQLGGEE-ALIPVYCYKDANVIKSGGIEILGLQAKSIAKK-KQRLEPVLEKYQFVPN- :  991
TcFAS XP_008200285.1 : LRGYDYDGVFRGIAESDNKGIAGKLKWSNNWISYIDTMLQFSILGQNTRELYLPTRLQRCVINPKEHLQYLNGLKE-E--DPSVYMYRNIGVIKSGGIELRGMKASLAPRRQQAQSPPKLEKYQFVPFE : 1133
AiFAS AGR49310.1       : LRGYNYGGIFRGIRASDPRGTCGELAWDDNWISFMDTMLQFGIIGVDTRELYLPTRLQRALIDPAAQLAAVAALGEGG--TLPVRMHRDIDVISAGGIEFRGVKTSLAPRRANPQNAPKLEKYVFLPYD : 1132
ApFAS XP_008187030.1 : LRGYNYKGMFRSLNRVNLDATVGRVGWFNNWVAFMDNMLQIQILKEDTRALFVPTSLQKLFINVKKHATILQTLPEDK-PEFPVYVYPEIDLIQSGGVEIRGLHANVIAKR-KPLADPVIEKYTFVPNI : 1151

CbFAS1 KU516006        : -------YTELSTADSVRVNAQIILEN-TLLRKIKSLEIIEDETLPEDYQVLSTILIDCLGDQPMIQPEAAIQT--------TAELNVDVTVINKKLVP---EDEYTLIIATNISKN--VKLLGEASNA : 1163
CbFAS2 KX467777        : NNQPLTEDSDKAKTHALTVLLQTVIENSSGALKLKVAEVGTDKPIE---AILAPTIKNILESEPMLSTDVTVVTG--GPVDTTSLEANDVKHVIRDVNSNTVAQDAHLVVASDLIMYDRRNVLDNAVAS : 1256
TcFAS XP_015836196.1 : -------STTLSLGDSIRVNTQIIIEN-SLESHFKSVEILDETSQ---LTPLSPLFCKSLDDVPVITPNVIISTK------KTLEEIPGVKIEDFVLTP---ESNLLLIIGTKLLQR--PTIPKLYFQA : 1098
TcFAS XP_008200285.1 : NNTALSENQDKAKTHALTVLLQTVLENSSGALKLKVAEVVSGRPVE---AILAPSVKDILEREPMLAVDASVVTS--DSIDSAALESSGVKVVHKDVSSNPIDQNLHLVVLTDALTHGKINLIENAANC : 1257
AiFAS AGR49310.1       : NAAVATEDTSRSKRDALTVSLQLVLEN-AGALKLKLAEAALDRPAE---ALLTPQALAVLESEPQVRVDATLAAGPTPAPYAAAVKDLGVKVLPKDGKSAPIESDCHLVMAADVLSRHGAATLEHLAAA : 1257
ApFAS XP_008187030.1 : I------ETDYTLEQIVRISLHLVIEN-LMDIKVKTVEILNKTFNP-DVQILSPIILDVLADLPLIQPEVSILSDG--THPQLKEIGSNITVEDKKLSS---DQSVLLTVGSGVLQD--SVLLEQVFAT : 1265

CbFAS1 KU516006        : LRENGFLICRESPDFENMP------QSIGLSVITVHRNPKETLVLLNKLSKKKK-TVFVDFTEDPQFDWLPKIQDKIKWAIKNK-EEIIIQSTKNSKSGLLGFVNCLRREPEGGIVKCVLVLDETE-DF : 1283
CbFAS2 KX467777        : LKPGGFILLEELKSPIDM----NKLDG--LELISKQLTDTKIYLLLRKPTEVPTDATVIQVTENN-FSWVEPLKDAMKQAESDG-RKIYLYVHGEELTGLVGMVNCLKQEPGGANLRSIFVQ-DSKAP- : 1375
TcFAS XP_015836196.1 : LSSKGFLISRESKDFHSEN------IEN-LEILTEFTTSDEKLIMFRKNCAIPV-PHFVKIGPET-FDWLPELQK----SLKNH-DSVIAFAQNETPEGILGLVSCLRPEPTGNKVRCYFLMDESP-EF : 1212
TcFAS XP_008200285.1 : IKDGGFVLLEEPKGAINS----GKLSTTELIVVSTQVTPTKSYVLLRKPVDVRSDSTAIQITENN-FAWVEPVKDAMKKSEGDG-SKIYLYVQGEELTGLVGMVNCLKQEPGGVGIRAVFIQ-DTNAP- : 1378
AiFAS AGR49310.1       : MADSGMLLLEEPHKALDDRAAQEMLQRAGLSPVSRQVAASCEYVLLRRAPALPAQHVVVDVADDTSYAWVDALREALARAEGED-MRVYCVAR-TPNSGVLGLCTCLRGEAGGRALRCYFLP-GAREPF : 1383
ApFAS XP_008187030.1 : LKPGGFLLTREKLNVEYS--------QGSVEVCLDASLEQERLMLVRKPLEQNI-VPIVVKISSTEFSWLPVLQKLLKADDASVSQKIVLVGEKDPKNGIVGLLNCIRKEPGGDRVRCVFIMDKCAPIF : 1385

CbFAS1 KU516006        : DPNVKLFADQLKKGLAINVYQNGSWGTYRHLLLK---KPEVVKSDHCFVAQTVRGDLSSLTWFEGSLNRDMLK--EPEKRIIKIHYSSLNFRDIMTASGKINVDAITTNRLEQECGQGFEFSGIDQSGQ : 1407
CbFAS2 KX467777        : KFSISAYENQLKKDLVHNVMKNNTWGCFRHIMLENYDDSGKLHVEHAYINTLTRGDLASLRWIEGPLGHYKGD--DADTELCHVYYAPLNFRDIMLATGKLPPDALPGDLAGQDCILGLEFSGRDSSGK : 1502
TcFAS XP_015836196.1: DPENSLFAAQIRKDLAFNVYKDGTWGTYRHLLLE---ELEEVESEHCYANVTVRGELGSLKWLEGPMRKDSVI--KKGNVMILNYYATINFKDVMLSSGRINVDVITSDRREQECAIGFEFAGRDLRGR : 1336
TcFAS XP_008200285.1 : KFSLSAYEQQLKKDLVHNVLKKNVWGSFRHILLDHDHDGGKLQVEHAYINTLTRGDLSSLRWIEGPLTYYKGG--DAKSELCHVYYAPLNFRDIMLATGKLPPDALPGDLAGQDCILGLEFSGRDSKGR : 1505
AiFAS AGR49310.1       : RPDAAPYAAQVRRDLAVNVLRAGVWGCYRHLPLG--DAEAQLQVEHAYVNTLTRGDLSSLRWIESPLRYARDVPQPARTDLCRVYCAPLNFRDIMLATGKLPPDALPGNLAGQECILGLEFSGRSSDGK : 1510
ApFAS XP_008187030.1 : SLDEPLYKKQLNKDLILNVYKNKVWGSYRHLLLE---PPSLIEVQHSCVNTSIRGDLSSLKWFQGSIVPFTKP--PAESDLVHVYYTALNFRDIMLATAKLAPEVIARGRINQESVIGFEYSGRRENGE : 1509
```

To continue the last alignment

```
CbFAS1 KU516006        : RVMGIIPHGALSTLAYADNHLLWRVPDEWSLAEAASVPVVYGTTVYALAIRGGMKRGDSILIHSGTGGVGQAAIRYALYHGCTVFTTVGNQAKRDFLKQLYPQLTDKNIGTSRDQSFVKMVMKRTKGRG : 1536
CbFAS2 KX467777        : RVMGMVAAKSLATTVLADPGFLWEVPEKWSLEEAATIPVVYGTSYYALVVRGGLKPGQSLLVHAGTGGVGQASISIALHMGCTVFTTVSSQAKRDFLKKTFPQLKDEHIGNSRDTSFEQLVMTQTNGRG : 1631
TcFAS XP_015836196.1 : RLCGMTDNSAFSSHISADPTLLWEIPDSWSLEDGATIPVVYGTVLYALLMVRKLRPGSSILIHSATGGIGLAAINVCLYYKCQIYLTVGTQEKRDYLRKMYPQIPQTRIGNSRDTSFEEMIKRETNGRG : 1465
TcFAS XP_008200285.1 : RVMGMVEARSLATTVIADTGFLWEVPEKWSLEEAATIPVVYGTSYYALIVRGGMKPGESILIHAGTGGVGQASIAIALHMGCKVFTTVSSQAKRDFLKKTFPQLNDDCIGNSRDTSFEQMVLTQTNGRG : 1634
AiFAS AGR49310.1      : RVMGMVAACGLASTVLADKGFLWEVPAKWSLEEASTVPVAYATAYYALAVRGRMQRGDSVLVHAGTGGVGQAAVAIALHAGCTVFTTVGTPDKRAFLRERFPTLPPENIGNSRDTSFEQLIKRRTRGRG : 1639
ApFAS XP_008187030.1 : RIMGMITSRALTNILVHDKYLAWKVPDSWSLEEAATVPVVYGTVIYALIVSGRMKRGDSVLIHAGTGGVGQAAITIALYYECEIFTTVGTAEKREFIKTHFPQIPESHIGNSRDSSFEQMIMMETDGKG : 1638

CbFAS1 KU516006        : VDIVLNSLAEDKLLASARCLGKGGRFLEIGKYDLFANSPLSLLLLEKEASFHGIVLDALFNSQP--SEKLTIYKLLGEGIRSGSIKPLGTTVFKTNEIEQAFRHMTRGIHMGKVLVEVQKEEKIETKKP : 1663
CbFAS2 KX467777        : VDCVLNSLAGSQLQASVRCLAIGGRFLEIGKVDLSNNSPLGMSIFLKNTTFHGILLDALFDSES--PEKRQVFNLVSEGIKTGAVRPLPKSVFNEHQVEQAFRFMASGKHIGKVLLQIREEESRKTQRP : 1758
TcFAS XP_015836196.1 : VDIVLNSLSEEKLMASVRCVAPGGRFLEIGKFDLANNTQLGLLMMEKGISYHGVMLDQIYNNSP--ELKRELSQIVSDGIKIGFIKPLPRVVFKREEIEAAHRYMMTGRHMGKVMLKIRHEEPKTAHIP : 1592
TcFAS XP_008200285.1 : VDLVLNSLANHQLQASVRCLAIGGRFLEIGKVDLSNNSPLGMSIFLKNTTFHGILLDALLDSDG--PDKREVVRLINEGIANGAVKPLPSTVFNENQTEQAFRFMASGKHIGKVVLKIRDEESRKAQRP : 1761
AiFAS AGR49310.1      : VDLVLNSLAADKLQASVRCLAEGGRFLEIGKLDLSNDTALGMSVFLKNTTFHGILLDALFDADSENSDKAAVVRCVTDGIAAGAVRPLPATVFSDHQLEQAFRYMATGKHIGKVVLRVRDEE-AAGARP : 1767
ApFAS XP_008187030.1 : VDMVLNSLAEEKLLASVRCLATGGRFLEIGKFDLANNSMIGMELFLKEISFHGVMLDTLFESPD--EWKEVFQRQVQENIDSGAIRPLVRTVFESDQVEPAFRYMAAGKHIGKVLIKIRDEEEDRFIKP : 1765

CbFAS1 KU516006        : D---IRGIPRYNCHADKCYIIVGGLGGLGLELTDWLILRGARKIVLTSRGGIRTGYQRSRIRLWRSYGVLVAISTSDVTTNDGCKELIEEANKLGPICAIFNLAAVLQDAAFENQNRKMYEISLAPKAY : 1789
CbFAS2 KX467777        : ATKTVAAIPRTYMDPEKSYVLVGGLGGFGLELAKWLVIRGATKIVLTSRSGVKTGYQSLCVRRWREKGIQVCISTADATTEKGAKRLLEEANALGPVGGIFNLAVVLRDAMMENQSEADFKTVCKPKVD : 1887
TcFAS XP_015836196.1 : NDQLFKVHPRFYANGSKVYIIIGGLGGVGLELADWLVLRGATKLVLVSRHGVTTGYQNQRLTIWKSYGVQVVISTHDITTKLGCLQLIKQAATMGPIDGIFNLGVVLKDALFEDQNEESFKLSRAPKST : 1721
TcFAS XP_008200285.1 : KPKTVTAIPRTYMDPDKSYVLVGGLGGFGLELANWLITRGASKIVLTSRSGIKTGYQSLCVRRWRDSGVKVLISTADATTEKGARRLLEEANGLGPVGGIFNLAVVLRDAMMENQTEADYKTACKPKVD : 1890
AiFAS AGR49310.1      : ASKLVSAIPRTYMHPAKSYVLVGGMGGFGLELAQWMVKRGCTRLVLNSRSGVRTGYQAWCVRRWREAGVRVLVSTADACSAAGARALLREAAALGAVGGVFNLAAVLRDAFLDKQTPADFQAVAKPKID : 1896
ApFAS XP_008187030.1 : RSINVKAYPRVTFDKNSSCIICGGLGGFGLELADWMVLRNSRNIILTTRTGIRNGYQALRKRVWESYGANVVISTADITTEEGVKQLLNEANKLGPVSTIFNLAVVLKDALFENQTEEDFKASFGPKAI : 1894

CbFAS1 KU516006        : ATKCLDEVSRSLCPELSDFVVFSSVSCGRGNPGQTNYGMANSVMERICEQRRRDGYPALAIQWGAVGEVGLVAEMQELHSELEISGTLPQRISSCMEVMDIALRQKSATIVSSMVVAEKRSSANSA--- : 1915
CbFAS2 KX467777        : GSKHLDAASRALAPHLDYFVNFSSVSCGRGNAGQANYGLANSAMERIAEARQAAGLPGIAIQWGAIGDVGLILETMGGN-DTEVGGTLPQRMSSCLSTMDIFLQQ-PHSVVASMVLAEKRKGTGSGSQV : 2014
TcFAS XP_015836196.1 : ATRFLDEITRVSCPELRHFVVFSSVACGRGNGGQTNYGMANSIMERICEKRKNKGFPALAIQWGAIGDVGLVAKMQKENRELVIGGTLQQKISSCLEVLDRFLKQ-GNPVVSSMVVAEKHNRNENL--- : 1846
TcFAS XP_008200285.1 : GSKQLDAASRSLAPHLDYFVNFSSVSCGRGNAGQSNYGMANSAMERICEARQAAGLPGVAIQWGAIGDVGLILETMGGN-DTEVGGTLPQRMASCLTTMDTFLQQ-PNAVVSSMVLAEKHKAGADANQV : 2017
AiFAS AGR49310.1      : ATKILDAATRELAPELEYFVVFSSVSCGRGNPGQSNYGLANSAMERIMEQRQADGLPGLAVQWGAIGEVGLIVETMGGD-ETVVGGTVPQRIASCMEALGALLAL-PHAVAASMVLADKRR-SAAAPQQ : 2022
ApFAS XP_008187030.1 : ATSLLDKYSRTMCPELKHFVIFSSVSCGRGNAGQTNYGMSNSVMERICEIRHSEGLPALAVEWGAVGEVGLVADMAEDNQEVVIGGTLQQKIGNCLEILDDLLTQKNNPIVSSMVVAEKRASSSNAG-- : 2021
```

To continue the last alignment

```
CbFAS1 KU516006       : SLSGTVLDIIGIKDIKSVSIQSTLAELGMDSMTGVEIRQVLEREFDVFLSAKELRTLTVARLMKLEEEK-QNQMTENKHN-------FSLKEIMSLLPTDNKGKAVILELESRISD-------TEGAPL : 2029
CbFAS2 KX467777       : SLTDAVGNILGIKDTSTVAPSATLADLGMDSLMGAEIKQTLERNYDLVLSAQEIRGLTFGHLLELSSGGSAGAPTETPGLQD------NN--LQFDKTAEIMPKKTLVQMPSKT-S-------DNKRTP : 2127
TcFAS XP_015836196.1 : SAVEAVALVLGIKDIKTVSQHATLAELGMDSMMGTEVIQLLEKEYEIYITAKDVRSLTFAKLREIEADRSEVKASDTKTS-------KGTNLLMKYIPDSKTSHLPIIRLESRVKE-------SANAPT : 1961
TcFAS XP_008200285.1 : SLIDAVANILGIKDASTVTASASLADLGMDSLMGAEIKQTLERNYDLVLNAQEIRALTFGKLTELGSGGAAPVETSAP-AGD------NQ--VQFDKNAEIMPSKCLVQMKSKG-G-------DSKKNP : 2129
AiFAS AGR49310.1       : DLLHAVANILGIKDPTKVSDSANLAELGMDSLMGAEIKQTLERGYDVVLGVQEIRGLTFSKLRGMAGGEDAAAGDAAPAATE------SADQVQFAALGELMPKQVLVKLPSAAPA-------GSELRP : 2138
ApFAS XP_008187030.1 : TIVDTVINILGLRDLKTISLHSTLAELGMDSMMAVEIKQTLERQFEVFLTPQDIRSMTFAKLQEIGSSDDKEKKADVDSKSIVQVPDVGLGYLIRAIGEESSAYNSVMRIPSLCEDGSVVEQPTTKLNT : 2150

CbFAS1 KU516006       : VFLIGGIEGYSEALRTLTRQLNARTISFQYDYDNPLDNIVKMAALYTSYIEDNITKDQPFCIVAYSYGICAGLEVVSVLESKGYKGTLVSIDGSPSVLQT-LASLIS--HENVK-IFEVSIIRETTNSY : 2154
CbFAS2 KX467777       : VFVLHPIEGVVKALEALAKNIDAPVYGLQCTATTPLTSIVDLAKHYIAQIKTVQPKG-PYVLIGYSFGACVAFEMGLWLEKNNEKVKLLLLDGSHSYVATHTGKAR--SKIQGGTAVEQSEVLVYFMMQ : 2253
TcFAS XP_015836196.1 : IFLLAGIEGVFHPLANLTKNLNAHIFGVQYDYNNPEDTITEVALKLLPHIEANLPQNRQFKIIGYSFGAAVGLEVVSLLEKMGLKGKMVTIDGSPSYLTASAKEYLP--EVDEA-EFQTALIFKILAVL : 2087
TcFAS XP_008200285.1 : VFVLHPIEGVVDALQVLASNIEAPVYGLQCTKDTPLTSIGDLAQYYNKQIKTVQPKG-PYTLVGYSFGACVAFEMGVQLENSGEQVKLLFIDGSPTYVATHTGKARNTKSLQGNTAAEQSEALVYFALQ : 2257
AiFAS AGR49310.1       : VFMVHPIEGVVELLRGVAAAVRAPVYGLQCTQAAPLEDMAALARHYVTHVRAMQPAA-PYTILGYSFGAGVAFEMALQLEQAGCETRLVLVDGSPAYVATHTTRGKK-KRTTRSAETDEADALAYFVQL : 2265
ApFAS XP_008187030.1 : LFVIPGLEGISSMLEPLSRNLNMQVLCLQYDILGTASTIEEMAATHYTLIQERLSADQEFSIVGYSFGGLIAIEVLKMLEKNNRTGKLWLIDSAPQFLKMTTELAIRGDKAQDQ-EIQVQLILRFLDLV : 2278

CbFAS1 KU516006       : VENDVISKYIPELMEIESLDERLERTADIVK-EYLPYEKEFIKSLYKGLIKRMWALKMYT-PTFTKINSKVILFKPTEQILKEYLYDLGLSLTCGNPVEVIEIEGNHLSILG---DENLANRINSLL--- : 2276
CbFAS2 KX467777       : FKDIDQQKVVSELASLKSWEERLTQAVQLLS-GATPYKAEEISDAATSFYGKLVAADKYT--PSGKFSGDVTLVKAIDNYVQM-GDDYGLSEVCKQKVNVESLKGNHRSILAGNSVEKIADILN-KLIKV : 2378
TcFAS XP_015836196.1 : VPIDLLVAFKDKLLKCKDMNERCDLVLEMLP-IEVEIKQKMEKQAALTLYKRFKAVIGYK-FEHEKIKSPVHLVKAKFAMVNE-EDDYQLSKCCES-VEFSTVNGDHGTILD---QEELVKKVEGFLL-- : 2208
TcFAS XP_008200285.1 : FKDIDQQKAVTELMSLKTWEERVARATQLVS-GAAPYPKEQLAAAAASFYYKLVAADKYK--PNMQFKGPVTLIRAIDNYVQM-GDDYGLSEVCKGKLKVQALEGNHRSILGGSTVQKIANIIHNDFIKA : 2383
AiFAS AGR49310.1       : FKDVDAAKVSSELERLPSWEARLAHTTALVGPAAGPHGAEALAAAANSFYRKLVIADTYK--PAGRLRAPVTLFTARDNYVTL-GEDYGLREVCAGELHTQQLAGTHRTILAGDAAAAIAQHLSQMLAH- : 2391
ApFAS XP_008187030.1 : WPHN-KTKITTELYQINSWDERLNYFIEQVP-DDVIYSKIYQKQLLNCAYIKLKALINYDGKPNGSLKTRTLLIRPTEQVLPI-SEDYGLSEYFNSAVMVHFVEGNHYSILE---NKKVAELISQSSSFKDL: 2404
```

Figure S1. Alignments of *C. bowringi* FAS protein sequences. Alignment was performed in MEGA 4.1 and Genedoc Version 2. *C. bowringi*, KU516006, KX467777; *Tribolium castaneum*, XP_015836196.1, XP_008200285.1; *Agrotis ipsilon*, AGR49310.1; *Acyrthosiphon pisum*, XP_008187030.1.

Figure S2

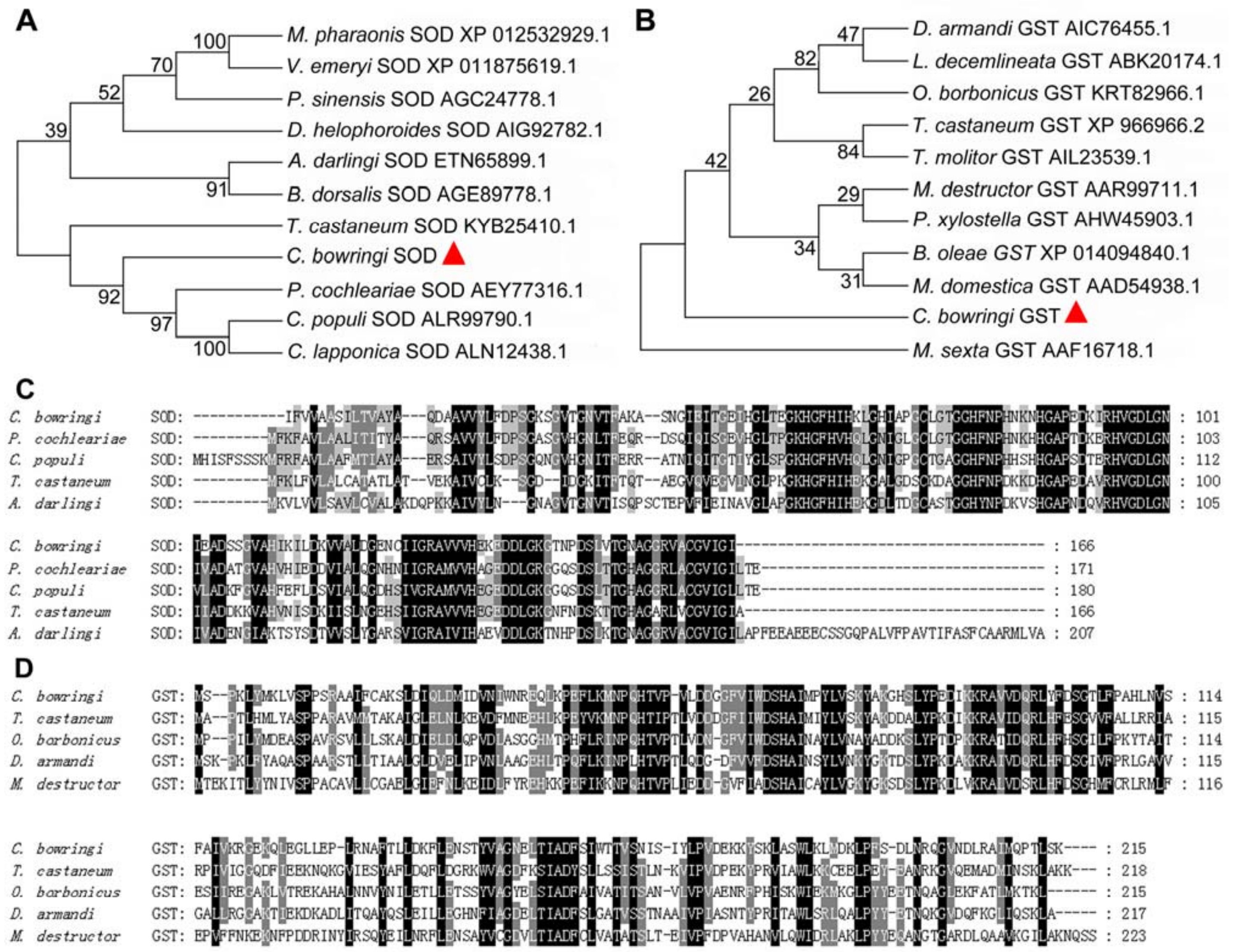

Figure S2. Gene identification of *C. bowringi SOD* and *GST*. Phylogenetic tree analysis of SOD (A) and GST (B). Red triangles denote *C. bowringi* SOD and GST proteins. Alignment was performed in MEGA 4.1 and Genedoc Version 2. The proteins of SOD are as followings, *Monomorium pharaonis*, XP 012532929.1; *Vollenhovia emeryi*, XP 011875619.1; *Pelodiscus sinensis*, AGC24778.1; *Dastarcus helophoroides*, AIG92782.1; *Anopheles darlingi*, ETN65899.1; *Bactrocera dorsalis*, AGE89778.1; *Tribolium castaneum*, KYB25410.1; *Phaedon cochleariae*, AEY77316.1; *Chrysomela populi*, ALR99790.1; *Chrysomela lapponica*, ALN12438.1. The proteins of GST are as followings, *Dendroctonus armandi*, AIC76455.1; *Leptinotarsa decemlineata*, ABK20174.1; *Oryctes borbonicus*, KRT82966.1; *Tribolium castaneum*, XP 966966.2; *Tenebrio molitor*, AIL23539.1; *Mayetiola destructor*, AAR99711.1; *Plutella xylostella*, AHW45903.1; *Bactrocera oleae*, XP 014094840.1; *Musca domestica*, AAD54938.1; *Manduca sexta*, AAF16718.1.

Figure S3

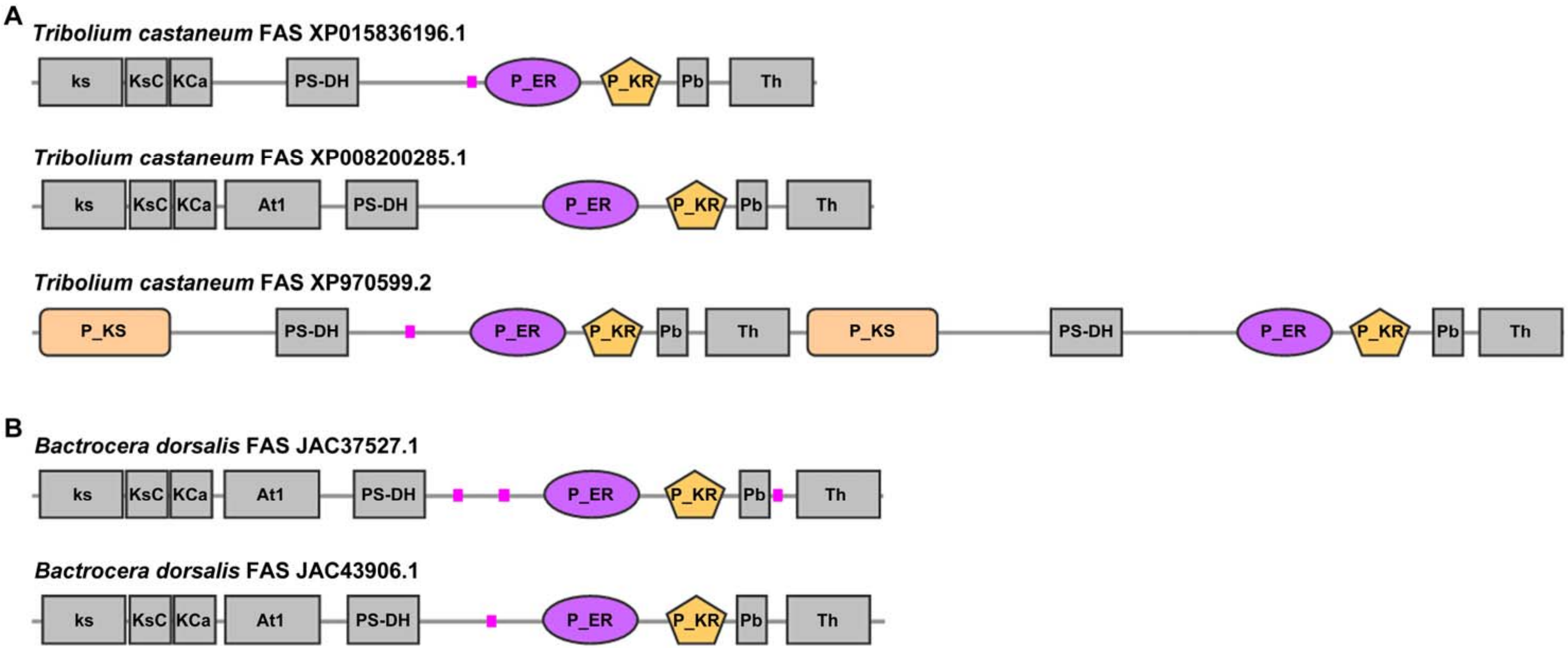

Figure S3. Schematic diagram of deduced domains of FASs of *Tribolium castaneum* (A) and *Bactrocera dorsalis* (B). *FASs* protein sequences were download from NCBI database and the protein domains were predicted with SMART tool. ks, ketoacyl-synt (pfam00109); KsC, Ketoacyl-synt_C (pfam02801); KCa, KAsynt_C_assoc (pfam16197); At1, Acyl_transf_1 (pfam00698); P_ER, PKS_ER (smart00829); P_KR, PKS_KR (smart00822); Pb, PP-binding (pfam00550); Th, Thioesterase (pfam00975); P_KS, PKS_KS (cd00833).

Table S1. Primers used in cloning *C. bowringi* cDNAs.

Primers	Sequences (5'-3')
FAS2-cloF	gttcctaacaggcaaaattg
FAS2-cloR	ttcggttccccaaatagaaaca
SOD-cloF	atattcgtggttgcagcttc
SOD-cloR	tattccgatgactccacagg
GST-cloF	atgagtcccaagttgtacatg
GST-cloR	cttggacaatgttggttgca
